# The correct and unusual coordinate transformation rules for electromagnetic quadrupoles

**DOI:** 10.1098/rspa.2017.0652

**Published:** 2018-05-09

**Authors:** J. Gratus, T. Banaszek

**Affiliations:** 1 Department of Physics, University of Lancaster, Lancaster LA1 4YB, UK; 2 Cockcroft Institute, Keckwick Lane, Daresbury WA4 4AD, UK

**Keywords:** tensor distributions, multipole expansions, derham currents, pre-metric electromagnetism, coordinate free approach, electric quadrupoles

## Abstract

Despite being studied for over a century, the use of quadrupoles have been limited to Cartesian coordinates in flat space–time due to the incorrect transformation rules used to define them. Here the correct transformation rules are derived, which are particularly unusual as they involve second derivatives of the coordinate transformation and an integral. Transformations involving integrals have not been seen before. This is significantly different from the familiar transformation rules for a dipole, where the components transform as tensors. It enables quadrupoles to be correctly defined in general relativity and to prescribe the equations of motion for a quadrupole in a coordinate system adapted to its motion and then transform them to the laboratory coordinates. An example is given of another unusual feature: a quadrupole which is free of dipole terms in polar coordinates has dipole terms in Cartesian coordinates. It is shown that dipoles, electric dipoles, quadrupoles and electric quadrupoles can be defined without reference to a metric and in a coordinates-free manner. This is particularly useful given their complicated coordinate transformation.

## 1. Introduction

Multipole expansions are used extensively as an approximation of extended particles where the mass or charge is considered to be concentrated at one point. Multipoles have been used in classical electrodynamics [1–3], quantum mechanics [4], as a model for polarization and magnetization [1,5–9], in determining the structure of molecules in chemistry [10–13] and recently in the idea of meta-atoms [14]. A distribution of charge can be approximated by a point charge together with a sum of moments [15]. The first correction is called the dipole, the second-order correction a quadrupole and so on. Three of the magnetic quadrupoles are identified as toroidal moments [14–17] and controversially called toroidal dipoles. The sources and potentials for electric and magnetic multipoles at rest (in flat space) are given in table 1 (see also [18]).

**Table 1.** Sources of static electric and magnetic dipoles and quadrupoles at the origin and their corresponding potential fields. Here *r*=∥***x***∥ and the components *γ*^*abc*^ satisfy the symmetry condition (2.3). Here *a*=0,1,2,3 and *μ*=1,2,3. Even in the static case, we can see that there are three electric dipoles, three magnetic dipoles, six electric quadrupoles and eight magnetic quadrupoles.

multipole	charge distribution	current distribution	number of components
electric monopole:	*ρ*_M_=*q* *δ*(***x***)	***J***_M_=**0**	1
electric dipole:	*ρ*_ED_=***p***_ED_⋅∇*δ*(***x***)	***J***_ED_=**0**	3
magnetic dipole:	*ρ*_MD_=0	***J***_MD_=***p***_MD_×∇*δ*(***x***)	3
electric quadrupole:	ρEQ=γ0μν∂2δ∂xμ∂x00ν	***J***_*EQ*_=**0**	6
magnetic quadrupole:	*ρ*_*MQ*_=0	JMQμ=γμνσ∂2δ∂xν∂x00σ	8
multipole	electric potential	magnetic potential	falloff of potentials as r→∞
electric monopole:	$\phi_{M}=\frac{q}{4\pi \epsilon_{0}r}$	***A***_M_=**0**	∼*r*^−1^
electric dipole:	*ϕ*_ED_=***p***_ED_⋅∇*ϕ*_M_	***A***_ED_=**0**	∼*r*^−2^
magnetic dipole:	*ϕ*_MD_=0	***A***_MD_=***p***_MD_×∇*ϕ*_M_	∼*r*^−2^
electric quadrupole:	$\phi_{\mathrm{EQ}}=\gamma^{0\mu \nu }\frac{\partial^{2}\phi_{M}}{\partial x^{\mu }\partial x^{\nu }}$	***A***_*EQ*_=**0**	∼*r*^−3^
magnetic quadrupole:	*ϕ*_*EQ*_=0	$A_{\mathrm{EQ}}^{\mu }=\gamma^{\mu \nu \sigma }\frac{\partial^{2}\phi_{M}}{\partial x^{\nu }\partial x^{\sigma }}$	∼*r*^−3^

**Figure 1.**
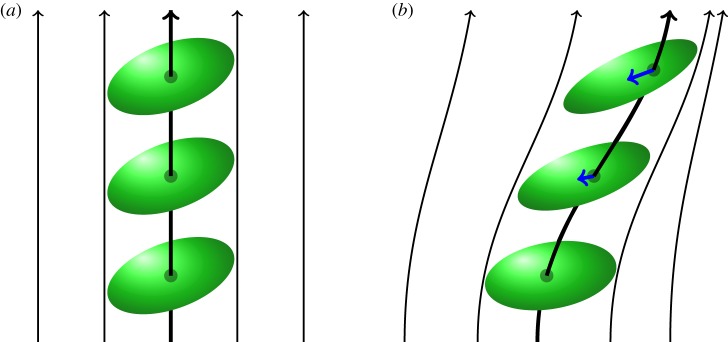
Flow of a quadrupole in coordinates adapted to the flow (*a*) and laboratory coordinates (*b*). Observe the appearance of a dipole (arrows) in the laboratory coordinate. Here quadrupoles are represented by ellipsoids, and dipoles by arrows. Clearly, the equations of motion are far simpler in the adapted coordinate system. (Online version in colour.)

What are the correct equations of motion for a quadrupole and higher-order moments? Although the equations of motion for the force and torque on a dipole are well established [1], the equivalents for quadrupoles is much less clear. One method is to consider that quadrupoles evolve due to a flow, as depicted in figure 1. The easiest method for analysing this quadrupole motion and evolution is to choose coordinate systems adapted to the flow, i.e. rectify the flow. In this coordinate system the quadrupole simply progresses unchanged as in figure 1*a*. One then needs to transform this equation into the laboratory coordinate system. For example to construct the equivalent of the Liénard–Wiechart fields [3], see figure 1*b*. However to do this, one needs the correct coordinate transformation rules. *The primary goal of this article is to establish the correct coordinate transformations*. This is important not only for transforming between an adapted and laboratory coordinate systems, but also between the spherical polars and Cartesian coordinates in flat space and also between the arbitrary coordinate systems in general relativity.

The components of a dipole transform in the familiar way as tensors. That is, using the Jacobian matrix, the entries of which are the partial derivatives of the coordinate transformation. One may naturally assume that the components of a quadrupole transform in a similar manner. Indeed this is the case if one limits oneself to Lorentz boosts and rotations in flat Minkowski space. Furthermore in such cases, a pure quadrupole, i.e. one that contains no dipole terms, would remain a pure quadrupole in all coordinate systems. However, with quadrupoles we have the following unusual properties:

(i) The coordinate transformations of quadrupoles require *the derivatives of the Jacobian matrix and an integral*. Although second derivatives of the coordinate functions are familiar for Christoffel symbols and jet bundles, those involving integrals have, as far as the authors are aware, never been considered before.
(ii) There is no such thing as pure quadrupole. The coordinate transformation of a quadrupole moment will, in general, produce a dipole moment.

Since the correct coordinate transformations for quadrupoles have been unknown up to now, the use of multipole expansions has been limited to Cartesian coordinates in flat space. This work, therefore will greatly expand the role of quadrupoles, so that they can be used to model extended charges in arbitrary coordinate systems and in arbitrary space–times. The tools developed in this article will enable researches to extend the results to higher-order multipoles.

With regards to point (ii) above, we give an example of a quadrupole which in polar coordinates has no dipole terms, whereas in Cartesian coordinates it does have dipole terms. As suggested in figure 1, the quadrupole which flows unchanged in the adapted coordinates gains a dipole in the laboratory frame. Although such a fluid flow is uncommon in electromagnetism, it is the natural Vlasov description for the dynamics of a distribution of charge in seven-dimensional phase–space–time. The extension of the coordinate transformations to seven dimensions can be easily handled using the coordinate-free approach detailed in this article. In the adapted coordinate system, one can also add additional forces, modelling internal collisions or self forces.

As well as the equation of motion for multipoles one may ask what are the electromagnetic fields for generated by them. The electromagnetic fields due to an arbitrary moving dipole, in flat Minkowski space–time, were first calculated by Ellis [2], and have been re-derived and re-expressed many times since [19–22]. The fields due to an arbitrary moving quadrupole or higher multipole were also derived by Ellis [3]. He derived the electromagnetic fields due to an arbitrary moving multipole in Minkowski space–time by differentiating the Liénard–Wiechart fields. As stated, these results require that the quadrupole is expressed in Cartesian coordinates. However, quadrupoles have been much less examined in the literature.

It is common to separate out dipoles into three electric and three magnetic dipoles. There is a choice, however, as to how to do this, one can separate them out with respect to the rest frame of the dipole [2] or with respect to a laboratory frame [20]. As the components of a dipole transform covariantly, these are easy to perform. One can also separate out the quadrupoles with respect to the rest frame or the laboratory frame. The complicated transformation rules, however, mean that these will mix with the dipoles under change of coordinates. Thus, the question of what is an electric or magnetic quadrupole in an arbitrary space–time is more subtle. We show that, with respect to the particle rest frame the electric quadrupole is well defined. As a result, multipoles form a natural hierarchy

1.1 $$\left\{\begin{array}{c} \text{Electric dipoles}\\ \left(\mathrm{Dim}=3\right) \end{array}\right\}\subset \left\{\begin{array}{c} \text{All dipoles}\\ \left(\mathrm{Dim}=6\right) \end{array}\right\}\subset \left\{\begin{array}{c} \text{Electric quadrupoles}\\ \left(\mathrm{Dim}=12\right) \end{array}\right\}\subset \left\{\begin{array}{c} \text{All quadrupoles}\\ \left(\mathrm{Dim}=20\right) \end{array}\right\}.$$

There is considerable interest in which aspect of electrodynamics can be defined without the use of a metric and hence without gravity [23]. If one relaxes the requirement that *τ* be proper time, then we see that monopoles, dipoles and quadrupoles do not require a metric for their definitions. Indeed even electric dipoles and electric quadrupoles can be defined without reference to a metric. By contrast, the magnetic multipoles require either a metric or a preferred coordinate system to define them. The advantage of such definitions are many fold:

— In general relativity, the stress-energy-momentum tensor can be derived by a variation of the metric in the Lagrangian. Knowing that multipoles are metric-free objects makes the variation much simpler.
— The definitions given mean that the concept of multipoles and electric multipoles can be generalized not only to higher dimensional space–times, but also to manifolds such as phase space or contact manifolds where there is no preferred metric. In particular, one can talk about multipole expansions of plasmas and beams of particles, where one takes moments of a probability distribution function in phase space.

When dealing with physical objects in arbitrary space–times, one has the choice either to define them with respect to a coordinate system and then give the coordinate transformations or to define them in a coordinate-free manner. Thus, it is perfectly acceptable to define quadrupoles using coordinates. However, such complicated transformations rules strongly promotes the coordinate-free definition of quadrupoles. In this article, we give such a coordinate-free definition.

This article is arranged as follows:

In §2, we present quadrupoles in the standard notation using coordinates and an integral over the worldline.

In §3, we derive the general coordinate transformation for quadrupoles. We also show which quadrupoles are in fact dipoles and which quadrupoles are electric dipoles.

In §4, we demonstrate a more abstract property of dipoles and quadrupoles, that is, that they can be defined without reference either to a coordinate system or to a metric.

Finally in §5, we conclude with some discussion and suggestion of future research. In appendix A, we prove some of the more technical statements from §4.

## 2. The standard representation of quadrupoles

In this article, the Greek indices *μ*,*ν*,*σ*=1,2,3 and the Latin indices *a*,*b*,*c*=0,1,2,3. We use the summation convention with implicit summation over pairs of matching high and low indices, unless otherwise stated.

The static electric and magnetic dipoles (table 1) can be combined into a single two-component antisymmetric tensor *γ*^*ab*^,

2.1 $$\gamma^{ab}+\gamma^{ba}=0$$

where

2.2 $$\gamma^{0\mu }=p_{\mathrm{ED}}^{\mu }\;\text{and}\;\gamma^{\mu \nu }=\epsilon^{\mu \nu \sigma }\;{\left(p_{\mathrm{MD}}\right)}_{\sigma }.$$

The quadrupoles components in table 1 can be combined into a single three-component object *γ*^*abc*^. Owing to conservation of charge, these satisfy the symmetry conditions:

2.3 $$\gamma^{abc}=\gamma^{acb}\;\text{and}\;\;\gamma^{abc}+\gamma^{bca}+\gamma^{cab}=0.$$

The symmetry conditions (2.3) give eight quadrupole components. These may be written *γ*^*μνν*^ for *μ*≠*ν* (no sum) which give six components and the pair (*γ*^123^, *γ*^231^). So that from (2.3) $\gamma^{\nu \mu \nu }=-\frac{1}{2}\gamma^{\mu \nu \nu }$ and *γ*^312^=*γ*^123^+*γ*^231^.

Toroidal ‘dipoles’ are given by

2.4 $$\gamma_{\mathrm{Tor}}^{\mu \nu \sigma }=T^{\nu }\delta^{\mu \sigma }+T^{\sigma }\delta^{\mu \nu }-2\;T^{\sigma }\delta^{\nu \sigma }.$$

Substituting (2.4) into ***J***_*MQ*_ (table 1) gives ***J***_*Tor*_=∇×∇×(***T****δ*). In our classification, these are considered quadrupole terms. In [14,16,17], these are actually referred to as toroidal ‘dipoles’. This despite the fact that 1: they involve the second derivative and 2: their potential fields fall off as *r*^−3^. In addition, they are not immune from the complicated transformations rules investigated in this article.

For moving multipoles, the sources given in table 1 have to be integrated over the worldline. In Minkowski space–time, the electromagnetic fields due to a moving electric charge are known as the Liénard–Wiechart fields. The source for the Liénard–Wiechart is the 4-current *J*^*a*^(*x*) which may be written in terms of the Dirac *δ*-function [1]

2.5 $$J_{M}^{a}(x)=q\int_{I}{\dot{C}}^{a}(\tau )\delta (x-C(\tau ))\;d\tau ,$$

where *C*^*a*^(*τ*) are the components of the worldline of the particle of charge *q*, ${\dot{C}}^{a}=C^{a}/\tau$ and *x* is a point in space–time. The parameter *τ* is usually considered to be the proper time of the particle, although it need not be, and the interval I⊂R is the range of *τ*. The source (2.5) is valid for any space–time, although in general the corresponding electromagnetic fields have not been calculated.

An arbitrary moving dipole in an arbitrary moving space–time may be written

2.6 $$J_{D}^{a}(x)=\int_{I}\gamma^{ab}(\tau )\frac{\partial \delta }{\partial x^{b}}(x-C(\tau ))\;d\tau ,$$

Multiple authors have found the electromagnetic field for a dipole in Minkowski space–time in terms of an integral of the retarded Green’s function.

Owing to conservation of charge the parameters defining the dipole are constrained to be antisymmetric (2.1) giving six components. However, once (2.1) is imposed the components *γ*^*ab*^=*γ*^*ab*^(*τ*) may be arbitrary functions of *τ*. It is easy to show that under a change of basis the *γ*^*ab*^ transforms as a tensor, given by (3.9). This is true both for global linear transformations, in Minkowski space–time, $x^{a}\to {\hat{x}}^{a}=A_{b}^{a}x^{b}$ and for local coordinate transformations $x^{a}\to {\hat{x}}^{a}={\hat{x}}^{a}(x^{0},\ldots ,x^{3})$.

The generalization of (2.6) to quadrupoles is less common. Kaufmann [24] was the first to express the quadrupole as an expansion to the second derivative of the *δ*-function. Ellis [2,3] observed that these can be written as

2.7 $$J_{Q}^{a}=\frac{1}{2}\int_{I}\gamma^{abc}(\tau )\frac{\partial^{2}\delta }{\partial x^{b}\partial x^{c}}(x-C(\tau ))\;d\tau .$$

He also calculated the electromagnetic fields for arbitrary moving quadrupoles and higher-order moments in Minkowski space–time, in terms of the integral of the retarded Green’s function. The *γ*^*abc*^ subject to (2.2) give 20 independent quadrupoles components. Again the *γ*^*abc*^=*γ*^*abc*^(*τ*) may be arbitrary functions of *τ*.

We observe that the quadrupoles given in (2.7) can also contain dipole terms. This can be seen in the static case when *t*=*τ*=*x*^0^ and so

$$\int_{I}\gamma^{ab0}(\tau )\frac{\partial^{2}\delta }{\partial x^{b}\partial x^{0}}(x-C(\tau ))\;d\tau =-\int_{I}\frac{d\gamma^{ab0}}{d\tau }\frac{\partial \delta }{\partial x^{b}}(x-C(\tau ))\;d\tau .$$

Thus, the electromagnetic fields due to a general static quadrupole given in (2.7) will contain both terms that fall off as distance cubed and terms that fall off as the fourth power of distance. The 20 quadrupole components split into six dipole components and 14 dipolefree-quadrupole components.

Although for global linear transformations in Minkowski space–time $x^{a}\to {\hat{x}}^{a}=A_{b}^{a}x^{b}$ the components *γ*^*abc*^(*τ*) transformation tensorially, this is not true for general coordinate transformation. As stated, the rules for a general coordinate transformation requires a second derivative of the coordinate functions and an integral. These are given in (3.10)–(3.12). Perhaps, it is less surprising since (2.7) contains a second derivative and an integral. However, it is unexpected when contrasted with the dipole case, where the components transform as tensors. One problem with such a transformation is that it will give rise to an arbitrary constant of integration. Fortunately, this constant does not affect the resulting quadrupole, as the terms are subsequently differentiated. Likewise, it will not affect the corresponding electromagnetic fields in flat space–time.

A quadrupole which does not appear to contain any dipole terms, will in general, acquire dipole terms when one performs a change of coordinates. This contrasts with the monopole term (2.5) which does not mix with other multipole terms under change of coordinates. As a simple example, consider a quadrupole at rest given in axial cylindrical coordinates (*t*,*r*,*θ*,*z*) with *γ*^*abc*^=0 except *γ*^211^=−2*γ*^121^=−2*γ*^112^=2*κ*, where κ∈R, *κ*≠0 is a constant. As it contains no components with a 0 index, one may expect that in Cartesian coordinates (*t*,*x*,*y*,*z*) with x=rcos⁡θ and y=rsin⁡θ, it would not contain any dipole terms. Indeed if one were to assume that *γ*^*abc*^ transforms tensorially, then this would be the case. However, with the correct transformation rules, given in (3.10)–(3.12) below, even in this simple case gives rise to a dipole term. Writing ${\hat{\gamma }}^{ab}$ and ${\hat{\gamma }}^{abc}$ for the (dipole and quadrupole) components with respect to Cartesian coordinates, the dipole term is ${\hat{\gamma }}^{12}=\kappa$. As stated, this would give rise to an *r*^−2^ fall off for the potential. Furthermore, when expressed in terms of (2.7) the component ${\hat{\gamma }}^{012}=\kappa t+\kappa_{0}$ which grows indefinitely and contains an arbitrary constant *κ*_0_. Fortunately as stated above, these are differentiated away.

As with the monopole and dipole terms, one can define a quadrupole in an arbitrary space–time, using (2.7) with *γ*^*abc*^ subject to (2.3). In this case, the lack of a preferred coordinate system means that one cannot separate out the dipoles from the quadrupoles. Likewise since we are not in Minkowski space–time, one cannot in general, use the fall off of the corresponding electromagnetic fields to distinguish the terms either. Thus in this case there is no concept of a dipolefree-quadrupole.

## 3. Coordinate transformations of quadrupoles

As the multipoles involve Dirac *δ*-functions, it is necessary to integrate them with test functions in order to evaluate them. Recall, these test functions are smooth and have compact support. That is, they are infinitely differentiable and are non-zero only on a bounded region of space–time. We write these test functions as (*ϕ*_0_,…,*ϕ*_3_) which are components of a covector. Acting on *ϕ*_*a*_ we have from (2.5)–(2.7), it is easy to see that the monopole, dipoles and quadrupoles give

3.1 $$\begin{array}{rl} \int_{M}J_{M}^{a}\phi_{a}\;d^{4}x & =q\int_{I}{\dot{C}}^{a}(\tau )\;\phi_{a}{|}_{C(\tau )}\;d\tau , \end{array}$$

3.2 $$\begin{array}{rl} \int_{M}J_{D}^{a}\;\phi_{a}\;d^{4}x & =-\int_{I}\gamma^{ab}(\tau ){\frac{\partial \phi_{a}}{\partial x^{b}}|}_{C(\tau )}\;d\tau \end{array}$$

3.3 $$\begin{array}{rl} \text{and}\;\int_{M}J_{Q}^{a}\phi_{a}\;d^{4}x & =\frac{1}{2}\int_{I}\gamma^{abc}(\tau ){\frac{\partial^{2}\phi_{a}}{\partial x^{b}\partial x^{c}}|}_{C(\tau )}\;d\tau , \end{array}$$

where *M* is space–time and we have assumed that *ϕ*_*a*_ is only non-zero on the coordinate patch (*x*^0^,…,*x*^3^). Given new coordinates $\left({\hat{x}}^{0},\ldots ,{\hat{x}}^{3}\right)$ then we require that *J*^*a*^_*M*_, *J*^*a*^_*D*_ and *J*^*a*^_*Q*_ all transform as vectors. That is

3.4 $$\hat{J_{M}^{a}}=\frac{\partial {\hat{x}}^{a}}{\partial x^{b}}J_{M}^{b},\;\hat{J_{D}^{a}}=\frac{\partial {\hat{x}}^{a}}{\partial x^{b}}J_{D}^{b}\;\text{and}\;\hat{J_{Q}^{a}}=\frac{\partial {\hat{x}}^{a}}{\partial x^{b}}J_{Q}^{b}.$$

These transformation are automatic in (3.1)–(3.3) since we assumed *ϕ*_*a*_ transformed as a covector, i.e.

3.5 $${\hat{\phi }}_{a}=\frac{\partial x^{a}}{\partial {\hat{x}}^{b}}\phi_{b}.$$

We also wish to consider allowing different parametrization τ∈I and $\hat{\tau }\in \hat{I}$. For example, one may be proper time and the other laboratory time.

Thus, relating the new and old coordinates then (3.1)–(3.3) and (3.4), (3.5) imply

3.6 $$\begin{array}{rl} & \int_{I}{\dot{C}}^{a}(\tau )\phi_{a}{|}_{C(\tau )}\;d\tau =\int_{\hat{I}}{\dot{\hat{C}}}^{a}(\hat{\tau }){\hat{\phi }}_{a}{|}_{C(\hat{\tau })}\;d\hat{\tau }, \end{array}$$

3.7 $$\begin{array}{rl} & \int_{I}\gamma^{ab}(\tau ){\frac{\partial \phi_{a}}{\partial x^{b}}|}_{C(\tau )}\;d\tau =\int_{\hat{I}}{\hat{\gamma }}^{ab}(\hat{\tau }){\frac{\partial {\hat{\phi }}_{a}}{\partial {\hat{x}}^{b}}|}_{C(\hat{\tau })}\;d\hat{\tau } \end{array}$$

3.8 $$\begin{array}{rl} \text{and}\; & \int_{I}\gamma^{abc}(\tau ){\frac{\partial^{2}\phi_{a}}{\partial x^{b}\partial x^{c}}|}_{C(\tau )}\;d\tau =\int_{\hat{I}}{\hat{\gamma }}^{abc}(\hat{\tau }){\frac{\partial^{2}{\hat{\phi }}_{a}}{\partial x^{c}\partial {\hat{x}}^{b}}|}_{C(\hat{\tau })}\;d\hat{\tau }. \end{array}$$

The charge *q* associated with the monopole is invariant under coordinate transformation, which follows from (3.6). As stated in the Introduction, the coordinate transformation of dipole components *γ*^*ab*^ is tensorial, i.e.

3.9 $${\hat{\gamma }}^{ab}=\frac{\partial {\hat{\phi }}_{c}}{\partial {\hat{x}}^{d}}\frac{\partial {\hat{x}}^{d}}{\partial x^{b}}\frac{\partial {\hat{x}}^{c}}{\partial x^{a}}\frac{d\tau }{d\hat{\tau }}\gamma^{ab}$$

since

$$\begin{array}{rl} \int_{I}\frac{\partial {\hat{\phi }}_{a}}{\partial x^{b}}\;{\hat{\gamma }}^{ab}\;d\hat{\tau } & =\int_{I}\frac{\partial \phi_{a}}{\partial x^{b}}\gamma^{ab}\;d\tau =\int_{I}\frac{\partial {\hat{x}}^{d}}{\partial x^{b}}\frac{\partial }{\partial {\hat{x}}^{d}}\left(\frac{\partial {\hat{x}}^{c}}{\partial x^{a}}{\hat{\phi }}_{c}\right)\gamma^{ab}\;d\tau \\ & =\int_{I}\left(\frac{\partial {\hat{x}}^{d}}{\partial x^{b}}\frac{\partial {\hat{x}}^{c}}{\partial x^{a}}\frac{\partial {\hat{\phi }}_{c}}{\partial {\hat{x}}^{d}}+\frac{\partial^{2}{\hat{x}}^{c}}{\partial x^{a}\partial x^{b}}{\hat{\phi }}_{c}\right)\gamma^{ab}\;d\tau \\ & =\int_{I}\frac{\partial {\hat{x}}^{d}}{\partial x^{b}}\frac{\partial {\hat{x}}^{c}}{\partial x^{a}}\frac{\partial {\hat{\phi }}_{c}}{\partial {\hat{x}}^{d}}\gamma^{ab}\;d\tau =\int_{I}\frac{\partial {\hat{\phi }}_{c}}{\partial {\hat{x}}^{d}}\frac{\partial {\hat{x}}^{d}}{\partial x^{b}}\frac{\partial {\hat{x}}^{c}}{\partial x^{a}}\frac{d\tau }{d\hat{\tau }}\gamma^{ab}\;d\hat{\tau }. \end{array}$$

In contrast to the dipole, the coordinate transformation of the components *γ*^*abc*^ of the quadrupole is given by

3.10 $${\hat{\gamma }}^{\mathrm{def}}=\frac{d\tau }{d\hat{\tau }}(A_{a}^{d}A_{b}^{e}A_{c}^{f}\gamma^{abc}+P^{de}\;{\dot{C}}^{f}+P^{df}\;{\dot{C}}^{e}),$$

where

3.11 $$A_{b}^{a}={\frac{\partial {\hat{x}}^{a}}{\partial x^{b}}|}_{C(\tau )},\;A_{bc}^{a}={\frac{\partial^{2}{\hat{x}}^{a}}{\partial x^{c}\partial x^{b}}|}_{C(\tau )},\;{\dot{C}}^{e}=\frac{dC^{e}}{d\tau }$$

and

3.12 $$P^{de}(\tau )=\int^{\tau }\gamma^{abc}(\tau^{\prime })(A_{c}^{d}(\tau^{\prime })A_{ab}^{e}(\tau^{\prime })-A_{c}^{e}(\tau^{\prime })A_{ab}^{d}(\tau^{\prime }))\;d\tau^{\prime }.$$

The proof of (3.10) is as follows:

$$\begin{array}{rl} \frac{1}{2}\int_{I}\frac{\partial^{2}\phi_{a}}{\partial x^{c}\partial x^{b}}\gamma^{abc}\;d\tau & =\frac{1}{2}\int_{I}\frac{\partial }{\partial x^{c}}\left(\frac{\partial }{\partial x^{b}}\left(\frac{\partial {\hat{x}}^{d}}{\partial x^{a}}{\hat{\phi }}_{d}\right)\right)\gamma^{abc}\;d\tau \\ & =\frac{1}{2}\int_{I}\frac{\partial }{\partial x^{c}}\left(\frac{\partial^{2}{\hat{x}}^{d}}{\partial x^{a}\partial x^{b}}{\hat{\phi }}_{d}+\frac{\partial {\hat{x}}^{d}}{\partial x^{a}}\frac{\partial {\hat{x}}^{e}}{\partial x^{b}}\frac{\partial {\hat{\phi }}_{d}}{\partial {\hat{x}}^{e}}\right)\gamma^{abc}\;d\tau \\ & =\frac{1}{2}\int_{I}(\frac{\partial^{3}{\hat{x}}^{d}}{\partial x^{c}\partial x^{a}\partial x^{b}}{\hat{\phi }}_{d}+\frac{\partial {\hat{x}}^{e}}{\partial x^{c}}\frac{\partial^{2}{\hat{x}}^{d}}{\partial x^{a}\partial x^{b}}\frac{\partial {\hat{\phi }}_{d}}{\partial {\hat{x}}^{e}}+\frac{\partial^{2}{\hat{x}}^{d}}{\partial x^{a}\partial x^{c}}\frac{\partial {\hat{x}}^{e}}{\partial x^{b}}\frac{\partial {\hat{\phi }}_{d}}{\partial {\hat{x}}^{e}}\\ & \;+\frac{\partial {\hat{x}}^{d}}{\partial x^{a}}\frac{\partial^{2}{\hat{x}}^{e}}{\partial x^{b}\partial x^{c}}\frac{\partial {\hat{\phi }}_{d}}{\partial {\hat{x}}^{e}}+\frac{\partial {\hat{x}}^{d}}{\partial x^{a}}\frac{\partial {\hat{x}}^{e}}{\partial x^{b}}\frac{\partial {\hat{x}}^{f}}{\partial x^{c}}\frac{\partial^{2}{\hat{\phi }}_{d}}{\partial {\hat{x}}^{f}\partial {\hat{x}}^{e}})\gamma^{abc}\;d\tau \\ & =\frac{1}{2}\int_{I}\left((A_{c}^{e}\;A_{ab}^{d}+A_{b}^{e}\;A_{ac}^{d}+A_{a}^{d}A_{bc}^{e})\frac{\partial {\hat{\phi }}_{d}}{\partial {\hat{x}}^{e}}+A_{a}^{d}A_{b}^{e}A_{c}^{f}\frac{\partial^{2}{\hat{\phi }}_{d}}{\partial {\hat{x}}^{f}\partial {\hat{x}}^{e}}\right)\gamma^{abc}\;d\tau \\ & =\frac{1}{2}\int_{I}\left(S^{de}\frac{\partial {\hat{\phi }}_{d}}{\partial {\hat{x}}^{e}}+A_{a}^{d}A_{b}^{e}A_{c}^{f}\gamma^{abc}\frac{\partial^{2}{\hat{\phi }}_{d}}{\partial {\hat{x}}^{f}\partial {\hat{x}}^{e}}\right)d\tau , \end{array}$$

where

$$S^{de}=(A_{c}^{e}A_{ab}^{d}+A_{b}^{e}A_{ac}^{d}+A_{a}^{d}A_{bc}^{e})\gamma^{abc}.$$

However,

$$\begin{array}{rl} (A_{c}^{e}A_{ab}^{d}+A_{b}^{e}A_{ac}^{d})\gamma^{abc} & =A_{c}^{e}A_{ab}^{d}(\gamma^{abc}+\gamma^{acb})\\ & =2A_{c}^{e}\;A_{ab}^{d}\gamma^{abc} \end{array}$$

and

$$\begin{array}{rl} A_{a}^{d}A_{bc}^{e}\gamma^{abc} & =-A_{a}^{d}A_{bc}^{e}(\gamma^{cab}+\gamma^{bca})\\ & =-A_{b}^{d}A_{ca}^{e}\gamma^{abc}-A_{c}^{d}A_{ab}^{e}\gamma^{abc}=-2A_{c}^{d}A_{ab}^{e}\gamma^{abc}. \end{array}$$

Hence

$$S^{de}=2(A_{c}^{e}A_{ab}^{d}-A_{c}^{d}A_{ab}^{e})\gamma^{abc},$$

so that *S*^*de*^+*S*^*ed*^=0. From (3.12), *S*^*de*^=−2(*dP*^*de*^/*dτ*) giving

$$\begin{array}{rl} \frac{1}{2}\int_{I}S^{de}\frac{\partial {\hat{\phi }}_{d}}{\partial {\hat{x}}^{e}}\;d\tau & =-\int_{I}\frac{dP^{de}}{d\tau }\frac{\partial {\hat{\phi }}_{d}}{\partial {\hat{x}}^{e}}\;d\tau =\int_{I}P^{de}\frac{d}{d\tau }\frac{\partial {\hat{\phi }}_{d}}{\partial {\hat{x}}^{e}}\;d\tau =\int_{I}P^{de}{\dot{C}}^{f}\frac{\partial^{2}{\hat{\phi }}_{d}}{\partial {\hat{x}}^{f}\partial {\hat{x}}^{e}}\;d\tau \\ & =\frac{1}{2}\int_{I}(P^{de}{\dot{C}}^{f}+P^{df}{\dot{C}}^{e})\frac{\partial^{2}{\hat{\phi }}_{d}}{\partial {\hat{x}}^{f}\partial {\hat{x}}^{e}}\;d\tau . \end{array}$$

Thus,

$$\begin{array}{rl} \frac{1}{2}\int_{I}\frac{\partial^{2}{\hat{\phi }}_{d}}{\partial x^{e}\partial x^{f}}{\hat{\gamma }}^{def}\;d\hat{\tau } & =J_{Q}[\phi ]=\frac{1}{2}\int_{I}(P^{de}{\dot{C}}^{f}+P^{df}{\dot{C}}^{e}+A_{a}^{d}A_{b}^{e}A_{c}^{f}\gamma^{abc})\frac{\partial^{2}{\hat{\phi }}_{d}}{\partial {\hat{x}}^{f}\partial {\hat{x}}^{e}}\;d\tau \\ & =\frac{1}{2}\int_{I}\frac{d\tau }{d\hat{\tau }}(P^{de}{\dot{C}}^{f}+P^{df}{\dot{C}}^{e}+A_{a}^{d}A_{b}^{e}\;A_{c}^{f}\gamma^{abc})\frac{\partial^{2}{\hat{\phi }}_{d}}{\partial {\hat{x}}^{f}\partial {\hat{x}}^{e}}\;d\hat{\tau } \end{array}$$

which gives (3.10). □

As stated certain quadrupoles are in fact dipoles. Consider the quadrupole given by (3.3) with *γ*^*abc*^(*τ*) given by

3.13 $$\gamma^{abc}=p^{ab}{\dot{C}}^{c}+p^{ac}{\dot{C}}^{b},$$

where *p*^*ab*^=*p*^*ab*^(*τ*) and *p*^*ab*^+*p*^*ba*^=0. Note that these satisfy (2.3). The quadrupole *J*^*a*^_*Q*_ is in fact a dipole *J*^*a*^_*D*_ where

3.14 $$\gamma^{ab}={\dot{p}}^{ab},$$

where ${\dot{p}}^{ab}=dp^{ab}/d\tau$. This follows since substituting (3.3) into (3.13) gives

$$\begin{array}{rl} \frac{1}{2}\int_{I}(p^{ab}{\dot{C}}^{c}+p^{ac}{\dot{C}}^{b})\frac{\partial^{2}\phi_{a}}{\partial x^{c}\partial x^{b}}\;d\tau & =\int_{I}p^{ab}{\dot{C}}^{c}\frac{\partial^{2}\phi_{a}}{\partial x^{c}\partial x^{b}}\;d\tau =\int_{I}p^{ab}\frac{d}{d\tau }\left({\frac{\partial \phi_{a}}{\partial x^{b}}|}_{C(\tau )}\right)d\tau \\ & =-\int_{I}{\dot{p}}^{ab}\left({\frac{\partial \phi_{a}}{\partial x^{b}}|}_{C(\tau )}\right)\;d\tau . \end{array}$$

Hence by comparing with (3.2), we see that $J_{Q}$ contains only a dipole term with (3.14).

We can now demonstrate our example of the ‘dipolefree-quadrupole’ in axial cylindrical coordinates outlined in the Introduction. Let *x*^*a*^=(*t*,*r*,*θ*,*z*) be axial cylindrical coordinates and ${\hat{x}}^{a}=(t,x,y,z)$ be Cartesian coordinates, with transformation functions x=rcos⁡θ and y=rsin⁡θ. The transformation rules (3.11) are given by

$$\begin{array}{rl} & A_{b}^{a}=\delta_{b}^{a}\;\text{except}\;A_{1}^{1}=\frac{\partial x}{\partial r}=\cos\theta ,\;A_{1}^{2}=\frac{\partial y}{\partial r}=\sin\theta ,\\ & A_{2}^{1}=\frac{\partial x}{\partial \theta }=-r\sin\theta ,\;A_{2}^{2}=\frac{\partial y}{\partial \theta }=r\cos\theta \\ \text{and}\; & A_{bc}^{a}=0\;\text{except}\;A_{12}^{1}=A_{21}^{1}=-\sin\theta ,\\ & A_{12}^{2}=A_{21}^{2}=\cos\theta ,\;A_{22}^{1}=-r\cos\theta ,\;A_{22}^{2}=-r\sin\theta . \end{array}$$

Thus, the integrated in (3.12) corresponding to *P*^12^, the only non-zero dipole component, is given by

$$\begin{array}{rl} & \gamma^{abc}(\tau^{\prime })(A_{c}^{1}(\tau^{\prime })\;A_{ab}^{2}(\tau^{\prime })-A_{c}^{2}(\tau^{\prime })A_{ab}^{1}(\tau^{\prime }))\\ & \;=\gamma^{112}(A_{2}^{1}A_{11}^{2}-A_{2}^{2}A_{11}^{2})+\gamma^{121}(A_{1}^{1}A_{12}^{2}-A_{1}^{2}A_{12}^{2})+\gamma^{211}(A_{1}^{1}A_{12}^{2}-A_{1}^{2}A_{12}^{2})\\ & \;=\kappa . \end{array}$$

Hence *P*^12^=−*P*^21^=*κt*+*κ*_0_ where *κ*_0_ is an arbitrary constant of integration. Hence ${\hat{\gamma }}^{012}=\kappa t+\kappa_{0}$. Using (3.13) and (3.14), we see that *P*^21^ gives rise to a dipole component with ${\hat{\gamma }}^{12}=\kappa$.

With respect to a coordinate system, the dipole components {*γ*^01^,*γ*^02^,*γ*^03^} are electric and {*γ*^12^,*γ*^13^,*γ*^23^} are magnetic. In this article, we only consider splitting the electric and magnetic components with respect to the instantaneous rest frame of the particle. In an arbitrary coordinate system, the electric dipole $J_{\mathrm{ED}}$ may be written as

3.15 $$\gamma^{ab}=w^{a}{\dot{C}}^{b}-w^{b}{\dot{C}}^{a},$$

where *w*^*a*^(*τ*) transforms as a vector. Note that replacing *w*^*a*^(*τ*) with $w^{a}(\tau )+\xi (\tau ){\dot{C}}^{a}(\tau )$, for any scalar *ξ*(*τ*), does not change $J_{\mathrm{ED}}^{a}$.

In space–time, there is a preferred rest coordinate system, called the Fermi coordinates, about a worldline. For quadrupole, we say that electric dipoles are those which in the Fermi coordinate system have components with a zero, i.e. *γ*^0*ab*^,*γ*^*a*0*b*^,*γ*^*ab*0^. From (3.13), we see that these contain all the dipoles. There are six dipolefree-electric quadrupoles. Likewise the magnetic eight magnetic quadrupole components contain only *γ*^*μνρ*^, where Greek indices run over *μ*,*ν*,*ρ*=1,2,3.

It turns out that identifying the electric quadrupoles in an arbitrary coordinate system is easy. These become

3.16 $$\gamma^{abc}={\dot{C}}^{a}q^{bc}+{\dot{C}}^{a}q^{cb}-{\dot{C}}^{b}\;q^{ac}-{\dot{C}}^{c}q^{ab}.$$

The *q*^*ab*^(*τ*) have no restrictions, but the *γ*^*abc*^(*τ*) is unchanged if we replace $q^{ab}(\tau )=q^{ab}(\tau )+s^{a}(\tau ){\dot{C}}^{b}(\tau )+s^{b}(\tau ){\dot{C}}^{c}(\tau )$ for any indexed scalars *s*^*a*^(*τ*). The *γ*^*abc*^ given by (3.16) satisfy the symmetry conditions (2.3). This gives the 12 independent electric quadrupole terms. If *q*^*ab*^+*q*^*ba*^=0, then (3.16) reduces to (3.13), with *p*^*ab*^=*q*^*ab*^. Equation (3.16) is proved in appendix A after we have introduced the coordinate-free and metric-free definitions.

## 4. Coordinate-free and metric-free definition of multipoles

As stated in the Introduction, in this section we introduce coordinate-free and metric-free definitions of dipoles, quadrupoles, electric dipoles and electric quadrupoles. This is because quadrupoles and electric quadrupoles are much easier to define in a coordinate-free manner. Let the set of all smooth *p*-form fields on space–time *M* be written *ΓΛ*^*p*^*M*. A test form is a form *ϕ*∈*ΓΛ*^*p*^*M* with compact support. The set of all test *p*-forms is written *Γ*_0_*Λ*^*p*^*M*.

Since the dipoles and quadrupoles are only non-zero along a worldline one must use notion of distributions in order to define them. Recall that the current 3-form, J, which includes dipoles and quadrupoles, is the source of Maxwell’s equations. In the language of exterior differential forms, Maxwell’s equations become

4.1 dF=0and dH=J,

where *F*∈*ΓΛ*^2^*M* is the electromagnetic 2-form encoding the electric fields ***E*** and the magnetic flux density ***B***, and where *H*∈*ΓΛ*^2^*M* is the excitation 2-form encoding the displacement field ***D*** and the magnetic field intensity ***H***. Here *d* is the exterior derivative. The fields *F* and *H* have to be related by constitutive relations. The constitutive relations for the vacuum are given by *H*=⋆*F*, where ⋆ is the Hodge dual, derived from the metric. These lead to the microscopic Maxwell equations, d⋆F=J. Taking the exterior derivative of the second equation in (4.1) leads to the continuity equation

4.2 dJ=0,

which in turn leads to conservation of charge. We say a J which satisfies (4.2) is **closed**.

Since Maxwell’s equations are linear, one can consider distributional currents. Following Schwartz, we define a distribution that is done on a test (4−*p*)-form *ϕ*∈*ΓΛ*^4−*p*^*M*. A test (4−*p*)-form has compact support. If *α*∈*ΓΛ*^*p*^*M* is a smooth *p*-form, we can construct a regular distribution *α*^*D*^ via

4.3 $$\alpha^{D}[\phi ]=\int_{M}\phi \wedge \alpha .$$

The definition of the wedge product, Lie derivatives, internal contraction and exterior derivatives on distributions are defined to be consistent with (4.3). Thus, for a distribution *Ψ* we set

4.4 $$\begin{array}{rl} \left(\Psi_{1}+\Psi_{2})[\phi \right] & =\Psi_{1}[\phi ]+\Psi_{2}[\phi ],\;(\beta \wedge \Psi )[\phi ]=\Psi [\phi \wedge \beta ],\;(d\Psi )[\phi ]={\left(-1\right)}^{\left(3-p\right)}\Psi [d\phi ],\\ \left(i_{v}\Psi )[\phi \right] & ={\left(-1\right)}^{\left(3-p\right)}\Psi [i_{v}\phi ]\;\text{and}\;(L_{v}\Psi )[\phi ]=-\Psi [L_{v}\phi ] \end{array}\}$$

Thus, for J to be closed requires

4.5 J[dλ]=0,

for all test forms *λ*∈*Γ*_0_*Λ*^0^*M*.

The monopole current $J_{M}$ is defined in terms of the worldline C:I→M where I⊂R is the domain of the parameter *τ*,

4.6 $$J_{M}[\phi ]=q\int_{I}C^{⋆}(\phi ),$$

where $C^{⋆}:\Gamma_{0}\Lambda^{1}M\to \Gamma_{0}\Lambda^{1}I$ is the pullback. Conservation of charge $dJ_{M}=0$ implies *q* is constant. In a coordinate system, this becomes (2.5) and (3.1).

Higher-order multipoles may be constructed by acting on $J_{M}$ with the operations given in (4.4), and then ensuring that the resulting distribution is closed. Unlike the dipole/quadrupole relations where they mix, the monopole can be separated off. That is all multipoles may be written

4.7 $$J_{\text{total}}=J_{M}+J_{\mathrm{Monopole}\;\mathrm{Free}},$$

for some value of the charge *q*. We say that J is **monopole free** if

**Figure 2.**
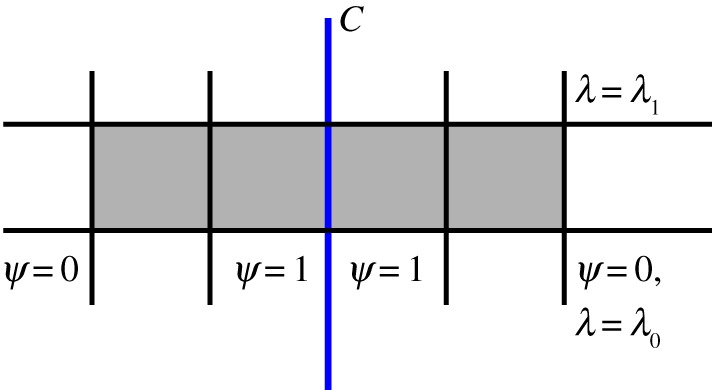
Construction of the test form *ψ* *dλ*∈*Γ*_0_*Λ*^1^*M*. Its support is the shaded region. (Online version in colour.)

4.8 J[ψ dλ]=0,

for all scalar fields *λ*,*ψ* such that *ψ* is flat in a neighbourhood of *C*, *C*^⋆^(*ψ*)=1 and the combination *ψ* *dλ* has compact support (figure 2). It is trivial to see that

4.9 $$J_{M}[\psi \;d\lambda ]=q\int_{I}dC^{⋆}(\lambda )=q(\lambda_{1}-\lambda_{0}),$$

where $\lambda_{1}=\lim_{\tau \to \sup(I)}\lambda (\tau )$ and $\lambda_{0}=\lim_{\tau \to \inf(I)}\lambda (\tau )$. We show in appendix A that J[ψ dλ] is independent of the choice of *λ*,*ψ* and hence (4.9) can be used to evaluate the charge associated with a multipole.

The **order of a multipole** is defined as follows. If

4.10 $$J[\lambda^{k+1}\phi ]=0\;\text{for all}\;\;\lambda \in \Gamma \Lambda^{0}M\text{ and }\phi \in \Gamma_{0}\Lambda^{1}M\;\text{such that}\;\;C^{⋆}(\lambda )=0$$

then we say that the order of J is at most *k*. Since we impose that *λ* vanishes on the image of *C*, this implies that we need to differentiate the argument *λ*^*k*+1^*ϕ* at least *k*+1 times for $J[\lambda^{k+1}\phi ]\neq 0$. We say dipoles have order at most one and quadrupoles have order at most two. Therefore, the terms in a dipole have at most one derivative, and those in a quadrupole at most two. This is consistent with the fact that the set of quadrupoles include all dipoles.

As stated the electric multipoles can be defined in a metric-free and coordinate-free manner, which contrast with the magnetic multipoles. We say that J is an **electric** multipole of order at most ℓ if

4.11 $$J[\lambda^{\ell }\;d\mu ]=0\;\text{for all}\;\lambda ,\mu \in \Gamma \Lambda^{0}M\;\text{such that}\;C^{⋆}(\lambda )=C^{⋆}(\mu )=0.$$

Clearly if J satisfies (4.10) at order *k*, then it satisfies (4.11) at order ℓ=*k*+1. Hence all dipoles are electric quadrupoles. In appendix A, we show that if J satisfies (4.11) at order ℓ then it also satisfies (4.10) at order *k*=ℓ. Thus, all electric quadrupoles are quadrupoles.

For a dipole at rest, not satisfying (4.11) with ℓ=1, i.e. $J_{D}[\lambda d\mu ]\neq 0$ for some *λ*,*μ* with *C*^⋆^(*λ*)=*C*^⋆^(*μ*)=0 then $J_{D}$ contains magnetic dipole components. Likewise if a quadrupole $J_{Q}$ does not satisfy (4.11) with ℓ=2, we say it has magnetic components.

Using (4.9)–(4.11), we can now define the multipoles we are interested in:

— A monopole, $J_{M}$ is a zero-order 3-form distribution over *C*.
— A dipole, $J_{D}$, is a closed, monopole-free, first-order 3-form distribution over *C*. This is equivalent to both (2.6) and (3.2).
— An electric dipole, $J_{\mathrm{ED}}$, is a dipole satisfying (4.11) with ℓ=1. This is equivalent to (2.6), (3.2) together with (3.15).
— A quadrupole, $J_{Q}$, is a closed, monopole-free, second-order 3-form distribution over *C*. This is equivalent to both (2.7) and (3.3).
— An electric quadrupole, $J_{\mathrm{EQ}}$, is a quadrupole satisfying (4.11) with ℓ=2. This is equivalent to (2.7), (3.3) together with (3.16).

These equivalences are all demonstrated in appendix A.

## 5. Conclusion and discussion

In this article, we have calculated the coordinate transformations associated with quadrupoles and their unusual property, namely second-order derivative and integration. There is always a tension as to the pro and cons of the using coordinate-free approaches. However given the complicated coordinate transformation given here, it is the opinions of the authors that the coordinate-free definition of quadrupoles is clearly justified. We have shown that electric multipoles are more ‘fundamental’ than magnetic multipoles since they can be defined without a metric or preferred coordinate system.

This work raises many interesting questions and directions one may pursue:

— As stated, using the metric one may define a pure magnetic dipole. That is, a dipole with no electric dipole terms. However, it is unknown to what extent one can define a magnetic quadrupole which does not contain any electric terms. Also unknown is whether one can define an electric quadrupole which does not contain magnetic dipole terms. By contrast, if one prescribes a laboratory coordinate system, then one can define all the objects: electric dipole (*dim*=3), magnetic dipole (*dim*=3), dipolefree electric quadrupole (*dim*=6) and dipolefree magnetic quadrupole (*dim*=8). As stated, these will mix with respect to other coordinate systems. It is natural to extend this analysis to higher-order multipoles. Raab & Lange [7] list the 77 electric terms up to octopole.
— It should be possible to extend this analysis to look at quadrupole sources for linearized gravity. This is important as a source for gravitational waves. In contrast to the closed 3-form for electromagnetic currents, the quadrupole in linearized gravity is a stress-energy-momentum tensor.
— As mentioned earlier, the results presented here can be extended not only to higher dimensions, but also to one- and two-dimensional sources, i.e. which trace out world-sheets and three-dimensional timelike manifolds. One can even construct an event multipole, which has support in just one event in space–time. One application of multipoles on higher-dimensional manifolds is in accelerator physics where the high-energy bunch of electrons can be expressed as a multipole expansion in seven dimensions (phase space + time).
— There is a longstanding debate in the literature about the correct equation of motion for a point charge that includes the back reaction, with most authors favouring the Abraham–Lorentz–Dirac equation [25–27]. This despite its well-documented pathologies. The problem for dipoles is more challenging as one would have to renormalize a force which goes as ∼*r*^−3^ as one approaches the dipole. Should that challenge be achieved and the radiation reaction for quadrupoles be desired then the prescription given here for the equations of motion will be needed. Alternatively, a higher-order theory of electromagnetism could be considered such as the Bopp–Podolski theory [28]. In this theory, the distributional sources for the moving multipoles would still be valid. Hence the electromagnetic fields due to a dipole would, we conjecture, grow as ∼*r*^−2^ as *r*→0 and hence may be renormalizable. Consequently, the quadrupole fields would grow as ∼*r*^−3^.
— The fact that the transformation rules for quadrupoles involve an integral poses the question about the bundle structure of quadrupoles. Since *γ*^*abc*^ is a function of *τ* one would look for a vector bundle over I, whose sections are in one-to-one correspondence with the set of quadrupoles. In future work [29], we show that such a vector bundle exists but is not unique and depends on a choice of thickening, that is a domain *U*⊂*M* and a map Π:U→I such that the combination *Π*(*C*(*τ*))=*τ*.

## Data accessibility

This article has no additional data.

## Appendix A. Statement and proof of the results in §sec4

Lemma A.1 The classification of dipoles and quadrupoles: *All dipoles* $J_{D}$*, i.e. closed, monopole-free, first-order 3-form distribution over *C* are given by (3.2).* *All quadrupoles* $J_{Q}$*, i.e. closed, monopole-free, second-order 3-form distribution over *C* are given by (3.3).*

Proof. Let $J_{D}$ be given by (3.2) and *λ*∈*ΓΛ*^0^*M* with *C*^⋆^(*λ*)=0. As there is only one derivative of *λ*^2^*ϕ*_*a*_ then $J_{D}[\lambda^{2}\phi ]=0$, hence $J_{D}$ has degree at most one. Likewise $J_{Q}$ given by (3.3) has degree at most two. By requiring (4.5), i.e. $J_{D}[d\lambda ]=0$ and $J_{Q}[d\lambda ]=0$ implies the symmetry conditions on *γ*^*ab*^ and *γ*^*abc*^, respectively. To show that a general dipole or quadrupole distribution with order *k* according to (4.10) can be written as (3.2) or (3.3), we need to consider an adapted coordinate system. Let (*z*^0^,*z*^1^,*z*^2^,*z*^3^) be a coordinate system adapted to the embedding *C*, so that *C*(*τ*)=(*τ*,0,0,0). By the manipulations in (4.4), we can see that the general multipole can be constructed from just addition, internal contraction, Lie derivatives and wedge products. Thus,

$$\Psi [\phi ]=\sum_{\mathrm{terms}\;\mathrm{like}}\int_{I}C^{⋆}(L_{v_{1}}\cdots L_{v_{r}}\phi )+\sum_{\mathrm{terms}\;\mathrm{like}}\int_{I}C^{⋆}(L_{v_{1}}\cdots L_{v_{r}}i_{w}\phi )\;d\tau .$$

For the dipole case then (4.10) implies $J_{D}[{\left(z^{\mu }\right)}^{2}\phi ]=0$ and $J_{D}[{\left(z^{\mu }+z^{\nu }\right)}^{2}\phi ]=0$ for *μ*=1,2,3 and hence $J_{D}[z^{\mu }z^{\nu }\phi ]=0$. Thus, there cannot be any terms with ∂^2^*ϕ*_*a*_/∂*z*^*μ*^∂*z*^*ν*^. Thus, the general degree three distribution of order at most one is given by

$$J_{D}[\phi ]=\int_{I}\left(\zeta^{\emptyset ,0}\phi_{0}+\sum_{\mu }\zeta^{\mu ,0}\partial_{\mu }\phi_{0}+\sum_{\nu }\zeta^{\emptyset ,\nu }\phi_{\nu }+\sum_{\mu ,\nu }\zeta^{\mu ,\nu }\partial_{\mu }\phi_{\nu }\right)d\tau ,$$

where ∂_*μ*_=∂/∂*z*^*μ*^. In this section (indexed), scalar fields (such as ∂_*c*_*ϕ*_0_) are implicitly evaluated on *C*(*τ*). Set $dJ_{D}=0$ and hence $J_{D}[d\lambda ]=0$ for all *λ*∈*Γ*_0_*Λ*^0^*M*. This gives the following equations:

A 1 $${\dot{\zeta }}^{\emptyset ,0}=0,\;{\dot{\zeta }}^{\mu ,0}-\zeta^{\emptyset ,\mu }=0\;\text{and}\;\;\zeta^{\mu ,\nu }-\zeta^{\nu ,\mu }=0,$$

where ${\dot{\zeta }}^{\mu ,0}=d\zeta^{\mu ,0}/d\tau$. The *ζ*^∅,0^=*q* gives the monopole term. The remaining terms are then given by

A 2 $$\begin{array}{rl} J_{D}[\phi ] & =\int_{I}\left(\sum_{\mu }(\zeta^{\mu ,0}\partial_{\mu }\phi_{0}-{\dot{\zeta }}^{\mu ,0}\phi_{\mu })+\sum_{\mu <\nu }\zeta^{\mu ,\nu }(\partial_{\mu }\phi_{\nu }-\partial_{\nu }\phi_{\mu })\right)d\tau \\ & =\int_{I}\left(\sum_{\mu }\zeta^{\mu ,0}(\partial_{\mu }\phi_{0}-\partial_{0}\phi_{\mu })+\sum_{\mu <\nu }\zeta^{\mu ,\nu }(\partial_{\mu }\phi_{\nu }-\partial_{\nu }\phi_{\mu })\right)d\tau . \end{array}$$

Thus, there are six free parameters. These correspond to the *γ*^*ab*^ for the adapted coordinates via *γ*^0*μ*^=−*ζ*^*μ*,0^ and *γ*^*νμ*^=−*ζ*^*μ*,*ν*^. Since there are six free parameters which is the same number in (3.2), then (3.2) covers all the dipoles. For quadrupoles, the general degree three distribution of order at most two is given by

$$\begin{array}{rl} J_{Q}[\phi ] & =\int_{I}(\zeta^{\emptyset ,0}\phi_{0}+\sum_{\mu }\zeta^{\mu ,0}\partial_{\mu }\phi_{0}+\sum_{\nu }\zeta^{\emptyset ,\nu }\phi_{\nu }+\sum_{\mu ,\nu }\zeta^{\mu ,\nu }\partial_{\mu }\phi_{\nu }+\sum_{\mu \leq \nu }\zeta^{\mu \nu ,0}\partial_{\mu }\partial_{\nu }\phi_{0}\\ & \;+\sum_{\mu \leq \nu }\sum_{\rho }\zeta^{\mu \nu ,\rho }\partial_{\mu }\partial_{\nu }\phi_{\rho })d\tau . \end{array}$$

Again setting $dJ_{Q}=0$ implies

$$\begin{array}{rl} \zeta^{\emptyset ,0} & =q,\;{\dot{\zeta }}^{\mu ,0}-\zeta^{\emptyset ,\mu }=0,\;\zeta^{\mu ,\mu }-{\dot{\zeta }}^{\mu \mu ,0}=0,\;\zeta^{\mu ,\nu }+\zeta^{\nu ,\mu }-{\dot{\zeta }}^{\mu \nu ,0}=0,\\ \zeta^{\mu \mu ,\mu } & =0,\;\zeta^{\mu \mu ,\rho }+\zeta^{\mu \rho ,\mu }=0\;\text{and}\;\zeta^{12,3}+\zeta^{13,2}+\zeta^{23,1}=0 \end{array}$$

for *μ*<*ν* and *μ*≠*ρ*. This gives

A 3 JQ[ϕ]=∫I(∑μζμ,0(∂μϕ0−∂0ϕμ)+12∑μ<ν(ζμ,ν−ζν,μ)(∂μϕν−∂νϕμ)+∑μ≤νζμν,0(∂μ∂νϕ0−12∂0∂νϕμ−12∂0∂μϕν)+∑μ≠νζμμ,ν(∂μ∂μϕν−∂ν∂μϕμ)+ ζ12,3(∂1∂2ϕ3−∂2∂3ϕ1)+ζ13,2(∂1∂3ϕ2−∂2∂3ϕ1)12)dτ.

Again the 20, non-monopole, parameters *ζ*^*ab*,*c*^ correspond to the 20 free parameters *γ*^*abc*^. These are given by

A 4 $$\begin{array}{rl} \gamma^{\mu \nu \rho } & =\zeta^{\nu \rho ,\mu },\;\gamma^{\nu \mu \mu }=\zeta^{\mu \mu ,\nu },\;\gamma^{0\mu \mu }=\zeta^{\mu \mu ,0}\\ \text{and}\;\gamma^{0\mu \nu } & =\zeta^{\mu \nu ,0},\;{\dot{\gamma }}^{\mu 0\nu }=\zeta^{\nu ,\mu },\;{\dot{\gamma }}^{\mu 00}=\zeta^{\mu ,0} \end{array}\}$$

for *μ*≠*ν*=1,2,3. Recall (2.3) implies *γ*^*abb*^=−2*γ*^*bab*^=−2*γ*^*bba*^. Observe that some of the components *γ*^*abc*^ are differentiated. □

Lemma A.2 The monopole term given by (4.9) is independent of *λ*,*μ*.

Proof. Substituting *ϕ*=*ψ* *dλ* into (A.2) and (A.3). We can use, for example,

$$\int_{I}\sum_{\mu <\nu }\zeta^{\mu ,\nu }(\partial_{\mu }(\psi \partial_{\nu }\lambda )-\partial_{\nu }(\psi \partial_{\mu }\lambda ))\;d\tau =\int_{I}\sum_{\mu <\nu }\zeta^{\mu ,\nu }(\partial_{\mu }\partial_{\nu }\lambda -\partial_{\nu }\partial_{\mu }\lambda )\;d\tau =0.$$

Similarly with the other terms. Thus, the only non-zero term is the monopole term. □

Lemma A.3 *For electric dipoles, the following definitions are equivalent*

— *Equation (4.11) with ℓ=1.*
— *Equation (3.15) in arbitrary coordinates.*
— *In adapted coordinates with *γ*^*μν*^=0 for all *μ*,*ν*=1,2,3.*

*For electric quadrupoles, the following definitions are equivalent*

— Equation (4.11) with ℓ=2.
— Equation (3.16) in arbitrary coordinates.
— In adapted coordinates with *γ*^*μνρ*^=0 for all *μ*,*ν*=1,2,3.

Proof. To show that (3.15) implies (4.11) with ℓ=1 we have.

$$\begin{array}{rl} J_{\mathrm{ED}}[\lambda \;d\mu ] & =\int_{I}(w^{a}{\dot{C}}^{b}-w^{b}{\dot{C}}^{a})\frac{\partial }{\partial x^{a}}\left(\lambda \frac{\partial \mu }{\partial x^{b}}\right)d\tau \\ & =\int_{I}(w^{a}{\dot{C}}^{b}-w^{b}{\dot{C}}^{a})\frac{\partial \lambda }{\partial x^{a}}\frac{\partial \mu }{\partial x^{b}}\;d\tau \\ & =\int_{I}\left(w^{a}\frac{dC^{⋆}(\mu )}{d\tau }\frac{\partial \lambda }{\partial x^{a}}-w^{b}\frac{dC^{⋆}(\lambda )}{d\tau }\frac{\partial \mu }{\partial x^{b}}\right)=0, \end{array}$$

since *C*^⋆^(*μ*)=*C*^⋆^(*λ*)=0. To show that (4.11) with ℓ=1 implies (3.15) then write $J_{D}$ in adapted coordinates (A.2). By acting on (*ψz*^*ρ*^ *dz*^*σ*^) where *ψ* has compact support, we have from (A.2)

$$\begin{array}{rl} J_{D}[\psi z^{\rho }\;dz^{\sigma }] & =\int_{I}\left(\sum_{\mu }\zeta^{\mu ,0}(\partial_{\mu }(\psi \;z^{\rho }\delta_{0}^{\sigma })-\partial_{0}(\psi \;z^{\rho }\delta_{\mu }^{\sigma }))+\sum_{\mu <\nu }\zeta^{\mu ,\nu }(\partial_{\mu }(\psi z^{\rho }\delta_{\nu }^{\sigma })-\partial_{\nu }(\psi z^{\rho }\delta_{\mu }^{\sigma }))\right)d\tau \\ & =\int_{I}2\zeta^{\rho ,\sigma }C^{⋆}(\psi )\;d\tau . \end{array}$$

Since this is true for all *ψ* we have *ζ*^*ρ*,*σ*^=0. Thus, we are left with three parameters *ζ*^*μ*,0^. Thus, both (3.15) and (4.11) are defined by three parameters and so they are equal. Since *γ*^*μν*^=*ζ*^*ν*,*μ*^ then the definitions in terms of adapted coordinates follows. The proof for quadrupoles is the same as the proof of dipoles. First substitute (3.16) into (4.11) with ℓ=2 to show that $J_{\mathrm{EQ}}[\lambda^{2}\;d\mu ]=0$. Then impose $J_{Q}[\lambda^{2}\;d\mu ]=0$ using $J_{Q}$ in adapted coordinates (A.3). This imply the eight terms $\zeta_{0}^{\mu \mu ,\nu }=\zeta_{0}^{12,3}=\zeta_{0}^{13,2}=0$ for *μ*≠*ν*=1,2,3. The remaining 12 terms give rise to the electric quadrupoles. □

Lemma A.4 *If* J *satisfies (4.11) at order ℓ then* J *also satisfies (4.10) at order *k*=ℓ.*

Proof. Let J satisfies (4.11) as order ℓ. By expanding out powers of the form $J[{\left(\lambda_{i_{1}}+\lambda_{i_{2}}+\ldots \right)}^{\ell }\;d\mu ]=0$ we can show $J[\lambda_{1}\lambda_{2}\cdots \lambda_{\ell }\;d\mu ]=0$ for all *λ*_1_,…,*λ*_ℓ_,*μ*∈*Γ*_0_*Λ*^0^*M* such that *C*^⋆^(*λ*_1_)=⋯=*C*^⋆^(*λ*_ℓ_)=*C*^⋆^(*μ*)=0. In adapted coordinates *ϕ*=*ϕ*_0_ *dτ*+*ϕ*_*μ*_ *dz*^*μ*^. So

$$\begin{array}{rl} J[\lambda^{\ell +1}\phi ] & =J[\lambda^{\ell +1}(\phi_{0}\;d\tau +\phi_{\mu }\;dz^{\mu })]\\ & =J[\lambda^{\ell }\phi_{0}\;d(\lambda \tau )]-J[\lambda^{\ell }\phi_{0}\tau \;d\lambda ]+J[\lambda^{\ell +1}\phi_{\mu }\;dz^{\mu }]\\ & =J[\lambda^{\ell -1}(\lambda \phi_{0})\;d(\lambda \tau )]-J[\lambda^{\ell -1}(\lambda \phi_{0}\tau )\;d\lambda ]+J[\lambda^{\ell }(\lambda \phi_{\mu })\;dz^{\mu }]=0. \end{array}$$
